# Growth Trajectories of Medication Adherence in Pediatric Organ Transplantation and Their Relationship to Posttransplant Health Outcomes

**DOI:** 10.1097/TXD.0000000000001907

**Published:** 2026-02-13

**Authors:** Michael O. Killian, Callie W. Little, Sonnie E. Mayewski, Schyler E. Brumm, Vir Hirani

**Affiliations:** 1 College of Social Work, Florida State University, Tallahassee, FL.; 2 Department of Behavioral Sciences and Social Medicine, College of Medicine, Florida State University, Tallahassee, FL.; 3 Florida Center for Reading Research, Florida State University, Tallahassee, FL.

## Abstract

**Background.:**

Nonadherence to immunosuppressive medication remains one of the most critical predictors of late acute rejection, hospitalization, and graft loss in pediatric organ transplant recipients. Despite its clinical importance, longitudinal patterns of adherence behavior in this population remain underexplored, particularly using objective pharmacokinetic indicators. This study aimed to examine adherence trajectories over time using the medication level variability index (MLVI), a validated biomarker of tacrolimus variability, and to assess their association with clinical and demographic factors.

**Methods.:**

We applied growth mixture modeling to longitudinal MLVI data from a large single-center sample of pediatric heart, liver, and kidney transplant recipients (N = 181). MLVI values were calculated from serial tacrolimus trough levels over a multiyear posttransplant period.

**Results.:**

Two adherence trajectories emerged with complete separation in MLVI (trajectory 1: range, 1.07–2.10; trajectory 2: range, 2.67–3.99). Across all years of follow-up, posttransplant hospitalization because of late acute rejection occurred in 33.4% of adherent versus 35.1% of nonadherent patients, and on a per-year basis 5.3% versus 7.7% of person-years, respectively (descriptively higher in the nonadherent trajectory). Public insurance status and patient functional status differed significantly between the 2 trajectories, suggesting social and structural determinants of adherence behavior.

**Conclusions.:**

These findings underscore the dynamic and heterogeneous nature of adherence across pediatric transplant populations. MLVI-based trajectory modeling offers a promising avenue for early risk identification and could inform the development of electronic medical record-integrated alerts or personalized adherence interventions. Future research should integrate psychosocial variables and expand to multicenter samples for enhanced generalizability.

## INTRODUCTION

Nonadherence to immunosuppressive medication remains 1 of the most significant predictors of late acute rejection (LAR) and poor posttransplant outcomes in pediatric patients.^1-3^ Rates of medication nonadherence range from 4% to 60% in samples of children and adolescents after organ transplantation, with several meta-analyses estimating an average of approximately 30%.^3-5^ Nonadherence can result in poor posttransplant outcomes including increased number and frequency of hospitalizations, need for biopsies testing for signs of organ rejection, episodes of rejection, and even mortality.^1-3,6,7^ Additionally, poor posttransplant health outcomes such as hospitalization and LAR increase stress and burden on patients and families and represent a loss of quality of life for these pediatric patients.^8^

Research has not adequately examined patient and family adherence to immunosuppressive medication longitudinally in pediatric organ transplantation. Several studies have measured adherence at only a few time points and reported adherence as a fluctuating and unstable phenomenon.^8-12^ A larger, multisite study of 400 liver transplant patients assessed medication adherence and reported similar instability >2 discrete time points (ie, 2 subsequent years).^10^ However, these studies used small samples, used indirect measures of adherence, and used limited statistical methods to model within-patient variability in adherence,^8,12,13^ all of which severely limit any conclusions we can draw from prior research. Additionally, the association between trajectories of adherence and important posttransplant outcomes has not been addressed.^10,14,15^

In response to the need for rigorous study of the longitudinal trajectories of adherence, we previously examined longitudinal data on patient medication adherence through multiple statistical approaches. Firstly, medication level variability index (MLVI) values^1,16,17^ across 332 patients were calculated every quarter using tacrolimus immunosuppressant medication trough levels.^18^ Multilevel modeling analyses revealed 35% of variation in MLVI values was among patients compared with only 8% of MLVI variation over time for patients (within-patient variability). Medication adherence was found to be relatively stable over time, yet older and male patients exhibited greater MLVI. Importantly, higher MLVI values predicted LAR and hospitalization in any given year.

Subsequent analyses using linear mixed-effects models (LMEMs) assessed change trajectories of MLVI and predictors of growth.^19^ Importantly, these analyses sought to examine the longitudinal growth patterns of MLVI change across patients and explore both linear and curvilinear growth. Results from LMEM analyses demonstrated an increase in adherence (ie, decrease in MLVI values) in the immediate years following transplantation yet was followed by limited patient changes in adherence in later years. Despite this trend, significant differences between individual patients remained a primary concern especially for those patients with greater MLVI scores and at higher risk for poor outcomes. Prior studies have similarly found relatively lower degrees of within-patient variation in adherence when compared with between-patient variation, although these findings were among smaller samples of patients.^13^

Across our prior work, demographic characteristics of patients predicted differences in MLVI and changes in MLVI over time. In the sample, older and male patients exhibited greater MLVI,^18^ yet age at transplant was not a significant predictor of change in MLVI over time suggesting the older patients remain most at risk for medication nonadherence. Female patients demonstrated more adherent growth patterns over time. Throughout, significant variation in MLVI values between patients remained.

The current study explored the number and nature of posttransplant growth trajectories of medication adherence in a large sample of pediatric heart, liver, and kidney transplant recipients from one of the largest pediatric transplant centers nationally. Growth mixture modeling (GMM) ^20-22^ was used to model trajectories of adherence accounting for both time-invariant (eg, sex) and time-variant (eg, current age) to describe group differences in longitudinal change. Most importantly, results from the GMM approach were hypothesized to identify multiple groups of patients who exhibit similar trajectories of growth in posttransplant adherence (ie, classes of individual growth trajectories or classes). Furthermore, we hypothesized that the likelihood of certain trajectories of growth using patient- and family-level characteristics and that differences between trajectories will predict likelihood of posttransplant outcomes. Examining individual trajectories of growth and using such trajectories will inform assessment in clinical work and trials, research on risk factors as stratified over time, and our ability to identify high-risk patients for targeted care or recruitment in adherence research.^10,14^

## MATERIALS AND METHODS

### Overview

Longitudinal patient and family data was collected and extracted from several sources within a large pediatric transplant program in the southwestern United States. Patient-level United Network for Organ Sharing (UNOS) data)^23^ was linked to transplant center electronic health records (EHRs) data through unique patient identification numbers. Trajectories of posttransplant medication adherence, patient and familial predictors, and posttransplant health outcomes were modeled using GMM. Posttransplant outcomes modeled were LAR with hospitalizations. University of Texas Southwestern Medical Center institutional review board reviewed and provided ethical approval for the study and waived the requirement for written informed consent.

### Participants and Sampling

The sample for the study (Table 1) were pediatric patients (ages 0 through 21) who received a kidney, heart, or liver transplant at the transplant center and completed at least 6 mo of follow-up. All transplant recipients from 2009 to 2017 were included in the sampling frame. Only a participant’s first transplant procedure was used. Participants were excluded from analyses if the patient moved during their first-year posttransplant, followed up with another transplant center during the first-year posttransplant, or failed to attend follow-up care.

**TABLE 1.** Descriptive statistic for MLVI, LAR status, and child demographics by class (n = 181)

Outcome	Total sample	Mean ± SD, n (%)		Test	Effect size
		Class 1, adherent (n = 167)	Class 2, nonadherent (n = 14)		
MLVI, mean ± SD	1.98 ± 1.94	1.46 ± 1.51	3.75 ± 2.77	U = 0^*b*^	Samples are fully disjointed
Children experiencing hospital LAR across all years, %	**63 (34.80%**)	**33.41%**	**35.14%**	**Fisher exact**	**OR = 0.91 (0.26-3.62**)
Hospital LAR status per year, %^a^	10 (5.52%)	5.33%	7.69%	Fisher exact	OR = 0.74 (0.09-34.92)
Child demographics					
Age at transplant, y, mean ± SD	4.95 ± 4.00	4.71 ± 3.73	4.94 ± 4.88	U = 5684.5	**δ** = –0.01 (–0.21 to 0.21)
Median age at transplant, y	4	3	2		
Range of age at transplant	0–17	0–17	0–16		
Sex, female, n (%)	95 (52.5%)	84 (50.30%)	4 (28.57%)	Fisher exact	OR = 2.97 (0.22-161.03)
Race, White, n (%)	141 (77.8%)	119 (71.23%)	11 (78.57%)	Fisher exact	OR = 0.55 (0.06-2.90)
Ethnicity, Latino, n (%)	81 (47.75%)	76 (45.51%)	5 (35.71%)	Fisher exact	OR = 1.27 (0.14-16.09)
Insurance status, public, n (%)	**57 (31.49%**)	**47(28.30%**)	**10 (70.58%**)	**Fisher exact**	**OR = 0.17**^a^ **(0.02-0.88**)
Life support, yes, n (%)	146 (80.66%)	138 (82.46%)	8 (60.20%)	Fisher exact	OR = 2.82 (0.41-15.67)
Functional status overall, mean ± SD	**9.28(1.65**)	**9.56 (1.27**)	**9.44 (2.05**)	**U = 11 984.0**	**δ = 0.34 (0.19-0.48**)
Organ type, n (%)					
Liver	46 (25.2%)	17 (10.24%)	4 (28.57%)		OR=0.29 (0.07-1.38)
Kidney	75 (41.6%)	**80 (47.9%**)	**2 (12.5%**)		**OR = 5.47**^a^ **(1.16-51.85**)
Heart	60 (33.2%)	69 (41.57%)	8 (57.14%)		OR = 0.53 (0.14-1.83)

Values in which participants were evaluated based on percentages.

a *P* < 0.05.

b *P* < 0.01.

LAR, late acute rejection; MLVI, medication level variability index; OR, odds ratio.

We compiled all tacrolimus trough levels obtained during routine care and calculated MLVI quarterly using the prior 12 mo of values, requiring ≥3 troughs in that window, consistent with our prior work.^18,19^ The analytic cohort for the GMM comprised 181 (54.7%) pediatric transplant recipients with 4 or more years of MLVI data, from whom 12 730 tacrolimus levels contributed to quarterly MLVI calculations. To contextualize observation density over time in the broader dataset, the full 332-patient cohort yielded 1470 valid patient-years of MLVI values.

### EHR Data

CMC began full adoption of a patient EHR system in 2009, and all eligible patients’ EHR data was extracted by research administration staff. The EHR data extraction included all blood assays of immunosuppressive medication and served as the measure of posttransplant medication adherence. The EHR data included demographic information for the patient and family (eg, age, age at transplant, sex, race/ethnicity, type of insurance, and family demographics and composition). Health and mental health diagnoses, dates of hospitalizations, reasons for hospitalizations, and transplant-related medical procedures were coded or calculated from EHR.

### Medication Level Variability Index

MLVI is the patient-level degree of variation in blood levels of tacrolimus calculated as each patient’s SD of tacrolimus levels per patient.^1,16,17^ Levels are obtained during regular clinical care and outpatient appointments. Higher MLVI scores indicate greater variability in blood levels and thus less consistent medication-taking. Studies of pediatric heart, liver, kidney, and lung transplant recipients have used this measure to assess medication adherence.^17,18,24-29^ Importantly, MLVI scores were found to predict LAR and hospitalization within larger studies of pediatric organ transplant recipients,^1,12,18^ further establishing the importance and predictive utility of MLVI. In the current study, MLVI was calculated quarterly using at least 3 tacrolimus trough blood levels as reported in prior research.^1,10,16,30^ In our prior work,^18^ increased monitoring and testing of patients did not significant impact MLVI values as the number of number of blood levels were only weakly and positively correlated with MLVI. As in other studies,^1,16^ we concluded that increased monitoring and testing of particular patients did not artificially increase MLVI values. Additionally, 2 prospective studies of adolescent heart transplant recipients demonstrated that MLVI was associated with dose-by-dose observed adherence behavior (ie, directly verified dose timing)^25^ and even interdose timing deviations.^31^ These analyses reinforce MLVI’s sensitivity to real-world medication behavior.

### UNOS Data

All transplant center UNOS data includes information on long-term health outcomes including episodes of LAR and hospitalization because of LAR. UNOS patient- and family-level data is collected when a patient is placed on a waiting list for transplantation, at the time of transplantation, and annually during posttransplant care. UNOS data includes variables related to pretransplant illness severity, transplant procedure, postoperative data, posttransplant complications, and health outcomes.

### Statistical Approach

GMM^32^ estimates possible trajectories of patients within the sample based on their differences in their initial status and longitudinal change between and within these otherwise unobserved trajectories of growth.^33^ We used GMM to capture distinct latent subgroups of medication adherence (ie, MLVI) behavior over time. While mixed-effects models can estimate average trends, GMM accommodates unobserved heterogeneity by identifying patient subgroups with differing trajectories, critical for a population where adherence is likely to vary meaningfully across individual patients. GMM identifies trajectories of growth by classifying patients exhibiting more similar types of growth across the posttransplant period when compared with other patients. Values of MLVI at each quarter posttransplant were used to estimate expected trajectories of growth in MLVI scores. GMM analyses estimated growth parameters that were allowed to vary across trajectories of growth. We explored the number of trajectories of growth using the cubic functional form of growth identified previously in the data.^19^ The baseline GMM analyses were conducted assuming all patients have the same pattern of growth in adherence (ie, 1 trajectory). We then followed an iterative process in which the estimated number of growth trajectories were increased. To determine the optimal number of trajectories of growth, we used several model fit indices recommended by Nylund et al^32^ and Muthén and Muthén.^34^ Model fit statistics included the Bayesian information criteria (BIC), values of entropy, and Vuong-Lo-Mendell-Rubin adjusted likelihood ratio test (VLMR-LRT). Lower values of BIC are preferred and indicate a better fitting model. Values of entropy represent the ability of a mixture model such as GMM to provide well separated trajectories.^35^ Entropy is high when the probability of cases belonging to 1 class is high. Higher entropy values (0.8 or above) indicate a better fitting model. The VLMR-LRT compares a model with X number of trajectories to a model with 1 fewer trajectory (X–1). A significant *P*-value for VLMR-LRT suggests that the model with fewer trajectories be rejected in favor of the model with X number of trajectories.^36^ All modeling was conducted in Mplus 8.11.^37^ Missing data was handled using the maximum likelihood estimation with a robust SEs estimator, which yields unbiased estimates when the pattern of missing data is completely at random.^38^ In our prior work to estimate longitudinal change in MLVI values, LMEM-determined growth and MLVI values did not significantly vary by organ type; thus, separate analyses were not conducted with subsets of patients by organ type.^14,18,19^ Additional analyses for differences between trajectories used Fisher exact test and Mann-Whitney U test, with calculated effect sizes.

## RESULTS

Across the 181 patients (Table 1) included in the GMM, a total of 12 730 tacrolimus troughs were available for quarterly MLVI computation. As expected in long-term follow-up, observation density declined with time since transplant. In the parent 332-patient cohort, the fraction of patients missing a yearly MLVI rose from 2.57% in year 1 to 87.71% in year 10, reflecting substantial attrition in later years.

Results of unconditional linear growth mixture models indicated differences in model fit dependent on the type of fit statistic considered (Table 2). We attempted to estimate unconditional cubic and quadratic growth mixture models, but the models either failed to converge, or produced warnings that the latent class covariance matrices were not positive definite. Because these models could not be estimated successfully: even with many random starts they failed to converge, produced abnormal termination messages, and the cubic term was essentially estimated as zero. We therefore retained the simpler linear model, which converged cleanly and was stable across replications (Table 3). We report the results of the unconditional linear models here. First, considering BIC values, the 2-trajectory model resulted in the lowest value (3458.303) followed by the 3-trajectory model (3467.325), then the 1-trajectory model (3486.651) indicating the 2-trajectory model as the best fitting model. Next, entropy values were highest for the 2-trajectory (0.93) model suggesting high probabilities that cases assigned to 1 trajectory were also low in probability of being assigned to any additional trajectory. Low BIC and high entropy values suggested the 2-trajectory model was the best fitting model to the data. Despite this, the VLMR-LRT was unable to reject a lower trajectory model in favor of the 2-trajectory model. Balancing these findings, the 2-trajectory model was selected as the best fitting model. Turning to the trajectory counts and trajectory proportions (represented in Table 1), for the final 2-trajectory model, the number of cases assigned to trajectory 1 was 167 and the number of cases assigned to trajectory 2 was 14. The average latent class probabilities for most likely trajectory membership were 0.99 for cases in trajectory 1 to be assigned to trajectory 1 and.01 for cases in trajectory 1 to be assigned to trajectory 2. The probability of cases in trajectory 2 to be assigned to trajectory 1 was 0.10 and for cases in trajectory 2 to be assigned to trajectory 2 was 0.90.

**TABLE 2.** Model fit statistics from the 1, 2, and 3 class growth mixture models

Model	Free parameters	AIC	BIC	Entropy	VLMR-LRT *P*
1 class	15	3438.591	3486.651	–	–
2 classes	**18**	**3400.631**	**3458.303**	**0.93**	**0.06**
3 classes	21	3400.041	3467.325	0.85	0.12

AIC, Akaike information criterion; BIC, Bayesian information criterion; VLMR-LRT, Vuong-Lo-Mendell-Rubin adjusted likelihood ratio test.

**TABLE 3.** Two-class linear model replicability

Run	STARTS (initial/final)	SEED	Best LL	BIC	Replicated?	Entropy	AvePP (by class)
(Linear 2-class)	2000/400	991329	–1682.316	3458.303	Yes^a^	0.930	0.902/0.988

Estimation terminated normally. Results showed stable EM progress with final-stage solutions converging to the same log-likelihood.

a Identical LL across final-stage solutions; normal termination. Model estimated with TYPE = MIXTURE, ESTIMATOR = MLR, ALGORITHM = EMA. Random starts were large (STARTS = 2000/400; OPTSEED = 991329).

AvePP, average posterior probability; BIC, Bayesian information criterion; EM, expectation–maximization algorithm; EMA, expectation–maximization with acceleration; LL, log-likelihood; MLR, maximum likelihood estimation with robust SEs.

Auxiliary information provided by predictors can indicate whether initial status and change trajectories across trajectories are differentially influenced. Once the number of trajectories was established using unconditional modeling, we attempted to include the covariates of sex and age as predictors of trajectory intercept and slope, but these models failed to converge. Huang et al^39^ have noted that the limitations of a conditional growth mixture model with a covariate typically involve increased complexity of model specification and the chance of occurrence of improper convergence because of a likelihood estimation problem.

**FIGURE 1.**
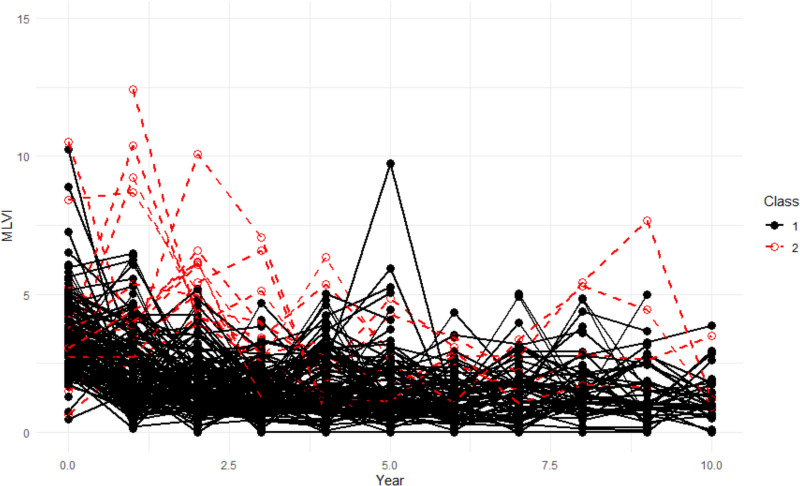
Plotted values of MLVI over time, by trajectory. MLVI, medication level variability index.

**FIGURE 2.**
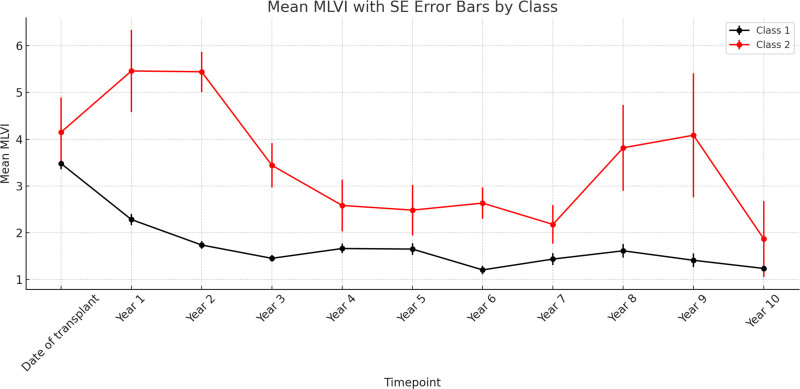
Mean MLVI with SE error bars by trajectory. MLVI, medication level variability index.

Trajectory assignment, trajectory probabilities, and the estimated means and variances for intercept and slope values from the GMM models were used for further analyses. Children in trajectory 1 had lower mean MLVI values than those in trajectory 2 (Figure 1). Trajectory membership was characterized by complete disjointness in MLVI values, with no overlap observed between the 2 groups. Patients in the adherent trajectory had MLVI values ranging from 1.07 to 2.10, whereas those in the nonadherent trajectory had values from 2.67 to 3.99, underscoring the absolute separation of the identified trajectories (Figure 2). Based on these findings, trajectory 2 was labeled as the significantly nonadherent trajectory with trajectory 1 demonstrating overall medication adherence. This finding underscores the distinct nature of the groups defined by our classification criterion.

Table 1 presents the participant demographic characteristics by adherence trajectory. Importantly, patients in the adherent trajectory had slightly lower rates of LAR than those in the nonadherent trajectory. Demographically, trajectories were composed of patients with similar age at transplant and racial identity. The nonadherent trajectory was composed of a significantly higher percentage of public insurance recipients (odds ratio [OR], 0.17; *P* = 0.02) with a significantly lower overall rating for functional status (U = 11 984; *P* < .001). Additionally, nonadherent trajectory higher percentage of males (OR, 2.97; *P* = 0.62), a slightly lower percentage of children identified as Latino (OR, 1.27; *P* = 0.99), and a lower percentage of prior life support status (OR, 2.82; *P* = 0.17), yet these differences were not statistically significant despite large effect sizes. Kidney transplants were the majority organ type for adherent trajectory, followed by heart transplants, but for nonadherent trajectory the largest percentage of transplants were heart, followed by liver.

## DISCUSSION

This study offers important insights into the trajectories of medication adherence in pediatric transplant patients by identifying distinct adherence trajectories using longitudinal person-centered modeling (ie, GMM). Our trajectory-based modeling captures this heterogeneity more appropriately and reveals how MLVI evolves over time in meaningful subgroups. This work supports the hypothesis that variability in adherence exists across pediatric transplant recipients,^1,8-11,18,19^ and that such variability is associated with differential posttransplant outcomes, including LAR and hospitalizations.^10,14,15,18,19^ The current study moves the adherence research literature beyond the simple application of MLVI to model trajectories of change over time. Results emphasize the continuous and dynamic nature of MLVI over time and its longitudinal association with LAR. The identification of 2 primary adherence trajectories emphasizes the need for tailored interventions and highlights opportunities to improve posttransplant care among high-risk patients.

The analysis revealed notable differences between the 2 trajectories in terms of mean MLVI values and clinical outcomes. Most importantly, patients in the adherent trajectory exhibited significantly lower average MLVI values and experienced lower rates of LAR compared with those in the nonadherent trajectory. Modeling of longitudinal trajectories with MLVI values identified an absolute separation of the trajectories with no overlapping MLVI values. Trajectories demonstrated complete separation in this sample between MLVI values of 2.10 and 2.67. The clear separation around an MLVI value of 2.1 highlights a potential clinical threshold for distinguishing adherence risk. This cutoff is data-driven and exploratory yet aligns closely with the ~2.0 threshold reported in prior multisite studies^1,30^ and used in study recruitment procedures to obtain samples of nonadherent patients.^1,10,25,30,40,41^ Importantly, the current study applied longitudinal averages across multiple MLVI assessments, in contrast to single-point cutoffs, yet the separation remained absolute.

A very important distinction in the current study is the complete separation around a MLVI of 2.10. The GMM model uses the average MLVI for patients across all their available MLVI data. This contrasts with the usual notion of a validated cutoff score at a single point in a patient’s care. Because there was no overlap of MLVI between trajectories, formal sensitivity and specificity analyses were redundant. Classification was effectively perfect at a threshold of approximately 2.1. This finding that may reflect sample size and single-center practice patterns and should not be interpreted as a universal diagnostic cut-point. Future multicenter studies should evaluate the generalizability, sensitivity, and specificity of this threshold across transplant care settings. Despite this, the current data and GMM analyses lend support to this threshold given the complete separation of 2 groups of patients even when using longitudinal MLVI values.

Although prior work with these data suggested that a cubic functional form best captured nonlinear change in MLVI values,^19^ our attempts to estimate quadratic and cubic growth mixture models failed to converge or produced improper solutions (eg, nonpositive definite covariance matrices). As a result, we restricted our final analyses to linear specifications. This simplification necessarily limits the nuance with which we can describe potential curvature in adherence trajectories. However, the absolute separation observed between trajectories, the high entropy values, and the stability of trajectory membership suggest that the linear model provided a robust and clinically meaningful classification. Moreover, recent simulation work has demonstrated that GMM is particularly sensitive to model complexity and sample size, with over-parameterized models prone to spurious solutions and instability.^42^ Thus, while future studies with larger, multicenter samples may be better positioned to test more complex growth forms, the linear specification in the present study offers a conservative and defensible representation of longitudinal adherence trajectories.

Patients in both trajectories were demographically similar in age at transplant and racial identity, differences emerged across other characteristics. Between-trajectory differences were significant for public insurance and functional status. Although not statistically significant, sex, ethnicity, and recipient of life support each varied between trajectories with large effect sizes. Female patients were found have lower MLVI values in the years following transplant,^18,19^ and results identified a greater portion of female patients within the adherent trajectory. Prior research has found African-American or minority patients and families at greater risk for nonadherence.^3,43,44^ Similarly, nonadherence has been found correlated with lower socioeconomic status including lower family income, receipt of public assistance, single-parent households, or public medical insurance.^3,29,45^ Receipt of life support during the pretransplant period has been found as positively associated with posttransplant adherence,^29^ and the current sample support these findings. Patient and family experience of severe health condition requiring transplantation may be traumatizing for families^46,47^ yet possibly contribute to posttraumatic growth.^48^ Post-traumatic growth may be a protective factor and a possible avenue for intervention in pediatric organ transplant care settings.

Results from the person-centered modeling has important implications for clinical assessment and practice. Our results suggest that adherence trajectories could be used by transplant teams to identify patients who would benefit from targeted adherence-promoting interventions, including personalized education and psychosocial support to address both promotion of and barriers to consistent medication use. Routine monitoring of MLVI levels may help in early identification of patients exhibiting higher variability, potentially allowing for proactive intervention before severe health consequences arise. The potential for GMM-derived trajectories of adherence to inform clinical decision-making and intervention design represents an innovative approach to assessment and management of chronic illness in children. Increasingly, care decision support systems have been developed to support diagnostics,^49^ prediction of health outcomes,^50,51^ and need for urgent intervention.^52^ Person-centered and longitudinal models of medication adherence could be integrated into these approaches to support multidisciplinary transplant teams in identifying high-risk behaviors. Future work should validate MLVI against larger samples with observed adherence and expand to multicenter studies to improve generalizability.

Limitations should be considered when interpreting the findings of this study. First, while the study uses longitudinal patient EHR data linked with administrative data from UNOS, the sample is drawn from a single pediatric organ transplant program in the southwestern United States. Although this center is 1 of the largest transplant programs nationally, the results may not by fully generalizable. Second, the study relied on all available patient tacrolimus blood levels recorded in EHR, which allowed for a comprehensive assessment of adherence trends over time using MLVI. However, patients had missing immunosuppressant medication blood levels in the later posttransplant period, leading to potential bias in the estimation of adherence trajectories.^18,19^ Previous modeling controlled for missing data and yielded similar results, suggesting minimal influence of missing data on key findings.

This study focuses on medication adherence as a key predictor of posttransplant health outcomes, yet the analysis does not account for other psychosocial factors that may influence adherence behavior. Psychosocial factors such as family stress, socioeconomic status, mental health conditions, and caregiver burden were not included in patient EHR or the UNOS data collection^53^ despite impacting adherence in pediatric patients.^8,14,53-55^ As we and others have noted previously, the absence of information on family stress, mental health, or social determinants of health in these data sources constrains any form of risk modeling in a more comprehensive way.^14,53^ For example, our inability to incorporate covariates such as age and sex into modeling may have been because of limited power to detect more nuanced subgroup differences among adherence trajectories. Alternative approaches may be available with larger, multicenter samples such as Bayesian estimation, 3-step procedures, or machine learning classifiers may offer more flexibly incorporate covariates and nonlinear growth forms into adherence trajectory modeling. Larger samples may also assist identifying clinically meaningful adherence trajectories within heterogeneous patient populations and inform tailored interventions for improving posttransplant outcomes.^56,57^

Applying GMM to MLVI trajectories has both practical and conceptual value. Although the trajectories identified in this study should be interpreted as exploratory and data-driven, rather than definitive diagnostic groups, they provide a promising foundation for hypothesis generation, risk stratification, and future clinical tools. Recent simulation and empirical work have demonstrated that GMM results are highly sensitive to measurement assumptions, particularly in longitudinal contexts where trajectory membership is latent and measurement precision may vary over time.^42^ At the same time, the use of the MLVI offers key advantages that bolster the clinical relevance of this modeling approach. MLVI is a well-established adherence proxy, validated across pediatric organ types.^1,14,17^ Its interpretation is clinically intuitive and has been consistently associated with poor outcomes.^1,12,18^ As such, modeling MLVI trajectories offers a rare opportunity to track medication adherence over time using data from routine care. In this context, the modeling serves not only as a statistical exercise but as a potential bridge between longitudinal adherence trajectories and actionable clinical insight.

This study offers novel insights into the longitudinal trajectories of medication adherence among pediatric transplant recipients by applying GMM to MLVI data. Two distinct adherence trajectories emerged: one characterized by consistent adherence, and another by persistently high variability. These findings reinforce that nonadherence is not a fixed or uniform behavior, but rather a dynamic, patient-specific process influenced by time and context. Demographic and clinical characteristics, including sex, race/ethnicity, public insurance, and pretransplant life support, differentiated these groups, highlighting the role of socioeconomic and psychosocial vulnerability in shaping adherence risk.

Importantly, these results support the clinical utility of MLVI trajectories as an early risk identification tool. The longitudinal model more accurately captures real-world variation and avoids the pitfalls of binary classification. Future work should expand this approach across multiple centers, integrate psychosocial predictors, and develop standardized, trajectory-informed adherence monitoring tools. Embedding MLVI-based risk stratification into routine transplant care may enable more personalized, proactive intervention ultimately to improve posttransplant outcomes.

## References

[1] Shemesh E Bucuvalas J Anand R. The medication level variability index (MLVI) predicts poor liver transplant outcomes: a prospective multi-site study. Am J Transplant. 2017;17:2668–2678. 28321975 10.1111/ajt.14276 PMC5607074

[2] Shemesh E Shneider BL Emre S. Adherence to medical recommendations in pediatric transplant recipients: time for action. Pediatr Transplant. 2008;12:281–283. 18331535 10.1111/j.1399-3046.2008.00920.x

[3] Oliva M Singh TP Gauvreau K. Impact of medication non-adherence on survival after pediatric heart transplantation in the U.S.A. J Heart Lung Transplant. 2013;32:881–888. 23755899 10.1016/j.healun.2013.03.008

[4] Dobbels F Ruppar T De Geest S. Adherence to the immunosuppressive regimen in pediatric kidney transplant recipients: a systematic review. Pediatr Transplant. 2010;14:603–613. 20214741 10.1111/j.1399-3046.2010.01299.x

[5] Kahana SY Frazier TW Drotar D. Preliminary quantitative investigation of predictors of treatment non-adherence in pediatric transplantation: a brief report. Pediatr Transplant. 2008;12:656–660. 18798360 10.1111/j.1399-3046.2007.00864.x

[6] Kelly DA. Current issues in pediatric transplantation. Pediatr Transplant. 2006;10:712–720. 16911496 10.1111/j.1399-3046.2006.00567.x

[7] Shemesh E Annunziato R Shneider B. Improving adherence to medications in pediatric liver transplant recipients. Pediatr Transplant. 2008;12:316–323. 18435607 10.1111/j.1399-3046.2007.00791.x

[8] Loiselle KA Gutierrez-Colina AM Eaton CK. Longitudinal stability of medication adherence among adolescent solid organ transplant recipients. Pediatr Transplant. 2015;19:428–435. 25879392 10.1111/petr.12480

[9] Lieber SR Shemesh E. Longitudinal stability of medication adherence: trying to decipher an important construct. Pediatr Transplant. 2015;19:348–350. 25940374 10.1111/petr.12484 PMC4429598

[10] Shemesh E Duncan S Anand R. Trajectory of adherence behavior in pediatric and adolescent liver transplant recipients—the MALT cohort. Liver Transpl. 2018;24:80–88. 28779546 10.1002/lt.24837 PMC5739966

[11] Massey EK Tielen M Laging M. Discrepancies between beliefs and behavior: a prospective study into immunosuppressive medication adherence after kidney transplantation. Transplantation. 2015;99:375–380. 25606787 10.1097/TP.0000000000000608

[12] Sakhuja S Himes R Carreker C. Impact of psychosocial factors on medication level variability index and outcomes in pediatric liver transplant recipients. Pediatr Transplant. 2023;27:e14425. 36325588 10.1111/petr.14425

[13] Wu YP Aylward BS Steele RG. Associations between internalizing symptoms and trajectories of medication adherence among pediatric renal and liver transplant recipients. J Pediatr Psychol. 2010;35:1016–1027. 20231258 10.1093/jpepsy/jsq014

[14] Killian MO Schuman DL Mayersohn GS. Psychosocial predictors of medication non-adherence in pediatric organ transplantation: a systematic review. Pediatr Transplant. 2018;22:e13188. 29637674 10.1111/petr.13188

[15] Lieber SR Helcer J Leven E. Pretransplant psychosocial risk factors may not predict late nonadherence and graft rejection in adult liver transplant recipients. Exp Clin Transplant. 2017;16:553–540. 10.6002/ect.2016.0349 28969524

[16] Christina S Annunziato RA Schiano TD. Medication level variability index predicts rejection, possibly due to nonadherence, in adult liver transplant recipients. Liver Transplant. 2014;20:1168–1177. 10.1002/lt.23930 PMC4177441 24931127

[17] Shemesh E Fine RN. Is calculating the standard deviation of tacrolimus blood levels the new gold standard for evaluating non-adherence to medications in transplant recipients? Pediatr Transplant. 2010;14:940–943. 20887400 10.1111/j.1399-3046.2010.01396.x PMC2992596

[18] Killian MO Little C Howry SK. Demographic factors, medication adherence, and post-transplant health outcomes: a longitudinal multilevel modeling approach. J Clin Psychol Med Settings. 2024;31:163–173. 37589865 10.1007/s10880-023-09970-4 PMC11487835

[19] Killian MO Little C Mayewski SE. Changes in medication adherence across the posttransplant period in pediatric organ transplant recipients. Clin Transplant. 2024;38:e15442. 39385672 10.1111/ctr.15442 PMC11469551

[20] Muthén BO Khoo S-T. Longitudinal studies of achievement growth using latent variable modeling. Learn Individ Diff. 1998;10:73–101.

[21] Nagin DS. Group-Based Modeling of Development. Harvard University Press; 2005.

[22] Nagin DS. Analyzing developmental trajectories: a semiparametric, group-based approach. Psychol Methods. 1999;4:139–157. 10.1037/1082-989x.6.1.18 11285809

[23] United Network for Organ Sharing. Data collection. Available at http://www.unos.org/data/about/collection.asp. Accessed March 27, 2010.

[24] Killian MO Triplett KN Mayersohn GS. Medication barriers and adherence: experiences of pediatric transplant recipients. Health Soc Work. 2022;47:165–174. 35771953 10.1093/hsw/hlac018 PMC12102065

[25] Killian MO Clifford S Lustria MLA. Directly observed therapy to promote medication adherence in adolescent heart transplant recipients. Pediatr Transplant. 2022;26:e14288. 35436376 10.1111/petr.14288

[26] Bilhartz JL Lopez MJ Magee JC. Assessing allocation of responsibility for health management in pediatric liver transplant recipients. Pediatr Transplant. 2015;19:538–546. 25824486 10.1111/petr.12466 PMC4485542

[27] de Oliveira JTP Kieling CO da Silva AB. Variability index of tacrolimus serum levels in pediatric liver transplant recipients younger than 12 years: non-adherence or risk of non-adherence? Pediatr Transplant. 2017;21:e13058. 10.1111/petr.13058 29034612

[28] Fredericks EM Dore-Stites D Well A. Assessment of transition readiness skills and adherence in pediatric liver transplant recipients. Pediatr Transplant. 2010;14:944–953. 20598086 10.1111/j.1399-3046.2010.01349.x PMC2951493

[29] Killian MO. Psychosocial predictors of medication adherence in pediatric heart and lung organ transplantation. Pediatr Transplant. 2017;21:e12899. 10.1111/petr.12899 28198130

[30] Shemesh E Mitchell J Neighbors K. Recruiting a representative sample in adherence research—the MALT multisite prospective cohort study experience. Pediatr Transplant. 2017;21:e13067. 10.1111/petr.13067 PMC5698095 28984072

[31] Killian MO Mayewski SE Brumm SE. Psychosocial considerations in pediatric heart transplantation: initial validation of the Pediatric Psychosocial Assessment Tool at a single center. JHLT Open. 2025;9:100319. 40687311 10.1016/j.jhlto.2025.100319 PMC12272104

[32] Nylund KL Asparouhov T Muthén BO. Deciding on the number of classes in latent class analysis and growth mixture modeling: a Monte Carlo simulation study. Struct Equ Model. 2007;14:535–569.

[33] Muthén B. Second-generation structural equation modeling with a combination of categorical and continuous latent variables: new opportunities for latent class–latent growth modeling. In: Decade of Behavior New Methods for the Analysis of Change. Collins LM Sayer AG, eds. American Psychological Association; 2001:291–322.

[34] Muthén B Muthén LK. Integrating person-centered and variable-centered analyses: growth mixture modeling with latent trajectory classes. Alcohol Clin Exp Res. 2000;24:882–891. 10888079

[35] Celeux G Soromenho G. An entropy criterion for assessing the number of clusters in a mixture model. J Classif. 1996;13:195–212.

[36] Lo Y Mendell NR Rubin DB. Testing the number of components in a normal mixture. Biometrika. 2001;88:767–778.

[37] Muthén LK Muthéén BO. Mplus User’s Guide. 8th ed. Muthén & Muthén; 1998–2018.

[38] Yuan K-H Bentler PM. 5. Three likelihood-based methods for mean and covariance structure analysis with nonnormal missing data. Sociolog Methodol. 2000;30:165–200.

[39] Huang D Brecht M-L Hara M. Influences of covariates on growth mixture modeling. J Drug Issues. 2010;40:173–194. 21841844 10.1177/002204261004000110 PMC3153912

[40] Killian MO Clifford SA Gupta D. Directly observed therapy to promote medication adherence in paediatric heart transplant recipients. Cardiol Young. 2021;31:2048–2050. 34092272 10.1017/S1047951121002109

[41] KillianMOMayewskiSEGuptaD. A multi-center randomized control trial of directly observed therapy to promote medication adherence in paediatric heart transplant recipients. Cardiol Young. 2024;34:1359–1362.38606638 10.1017/S1047951124000775

[42] Soland J Cole V Tavares S. Evidence that growth mixture model results are highly sensitive to scoring decisions. Multi Behav Research. 2024;60:487–508. 10.1080/00273171.2024.2444955 39812448

[43] Simons LE Gilleland J Blount RL. Multidimensional adherence classification system: initial development with adolescent transplant recipients. Pediatr Transplant. 2009;13:590–598. 18992064 10.1111/j.1399-3046.2008.01038.x

[44] Connelly J Pilch N Oliver M. Prediction of medication non-adherence and associated outcomes in pediatric kidney transplant recipients. Pediatr Transplant. 2015;19:555–562. 25917112 10.1111/petr.12479

[45] Davies RR Russo MJ Reinhartz O. Lower socioeconomic status is associated with worse outcomes after both listing and transplanting children with heart failure. Pediatr Transplant. 2013;17:573–581. 23834560 10.1111/petr.12117

[46] Annunziato RA Stuber ML Supelana CJ. The impact of caregiver post–traumatic stress and depressive symptoms on pediatric transplant outcomes. Pediatr Transplant. 2020;24:e13642. 31880384 10.1111/petr.13642

[47] Masood SS Triplett KN Killian M. Examining the association of medical complications and posttraumatic stress symptoms in pediatric solid organ transplant patients and their caregivers. Pediatr Transplant. 2021;25:e14030. 34076930 10.1111/petr.14030

[48] Triplett KN Mayersohn GS Masood SS. Posttraumatic growth in youth, young adults, and caregivers who experienced solid organ transplant. J Pediatr Psychol. 2021;47:965–977. 10.1093/jpepsy/jsab134 34957509

[49] Grigull L Lechner WM. Supporting diagnostic decisions using hybrid and complementary data mining applications: a pilot study in the pediatric emergency department. Pediatr Res. 2012;71:725–731. 22441377 10.1038/pr.2012.34

[50] Klann JG Anand V Downs SM. Patient-tailored prioritization for a pediatric care decision support system through machine learning. J Am Med Inform Assoc. 2013;20:e267–e274. 23886921 10.1136/amiajnl-2013-001865 PMC3861915

[51] Mani S Ozdas A Aliferis C. Medical decision support using machine learning for early detection of late-onset neonatal sepsis. J Am Med Inform Assoc. 2014;21:326–336. 24043317 10.1136/amiajnl-2013-001854 PMC3932458

[52] Lee EK Yuan F Hirsh DA. A clinical decision tool for predicting patient care characteristics: patients returning within 72 hours in the emergency department. Am Med Inform Assoc. 2012;2012:495–504. PMC3540516 23304321

[53] Killian MO Payrovnaziri SN He Z. Machine learning-based prediction of health outcomes in pediatric organ transplantation recipients: a study of single transplant center. JAMIA Open. 2021;4:ooab008. 34075353 10.1093/jamiaopen/ooab008 PMC7952224

[54] Dew MA Dabbs AD Myaskovsky L. Meta-analysis of medical regimen adherence outcomes in pediatric solid organ transplantation. Transplantation. 2009;88:736–746. 19741474 10.1097/TP.0b013e3181b2a0e0 PMC2769559

[55] Lefkowitz DS Fitzgerald CJ Zelikovsky N. Best practices in the pediatric pretransplant psychosocial evaluation. Pediatr Transplant. 2014;18:327–335. 24802341 10.1111/petr.12260

[56] Galatzer-Levy IR Ma S Statnikov A. Utilization of machine learning for prediction of post-traumatic stress: a re-examination of cortisol in the prediction and pathways to non-remitting PTSD. Transl Psychiatry. 2017;7:e1070–e1070. 10.1038/tp.2017.38 PMC5416681 28323285

[57] Galatzer-Levy IR Ankri Y Freedman S. Early PTSD symptom trajectories: persistence, recovery, and response to treatment: results from the Jerusalem Trauma Outreach and Prevention Study (J-TOPS). PLoS One. 2013;8:e70084. 23990895 10.1371/journal.pone.0070084 PMC3750016

